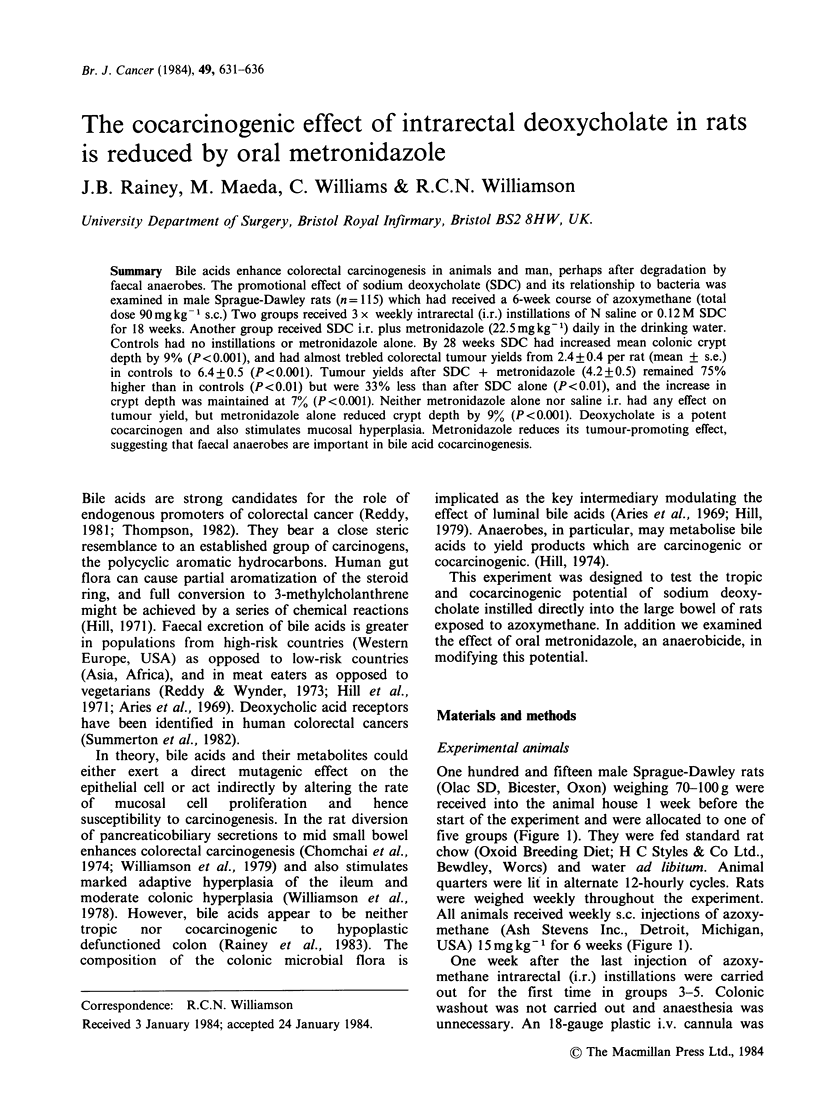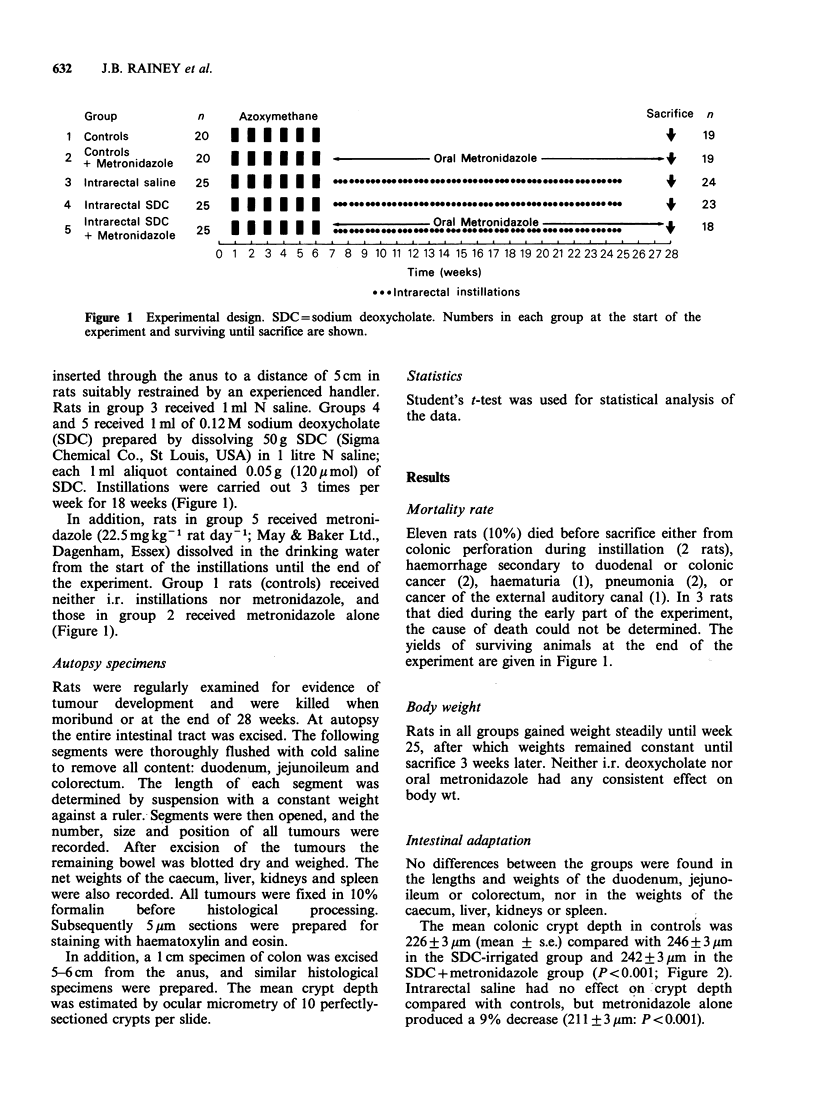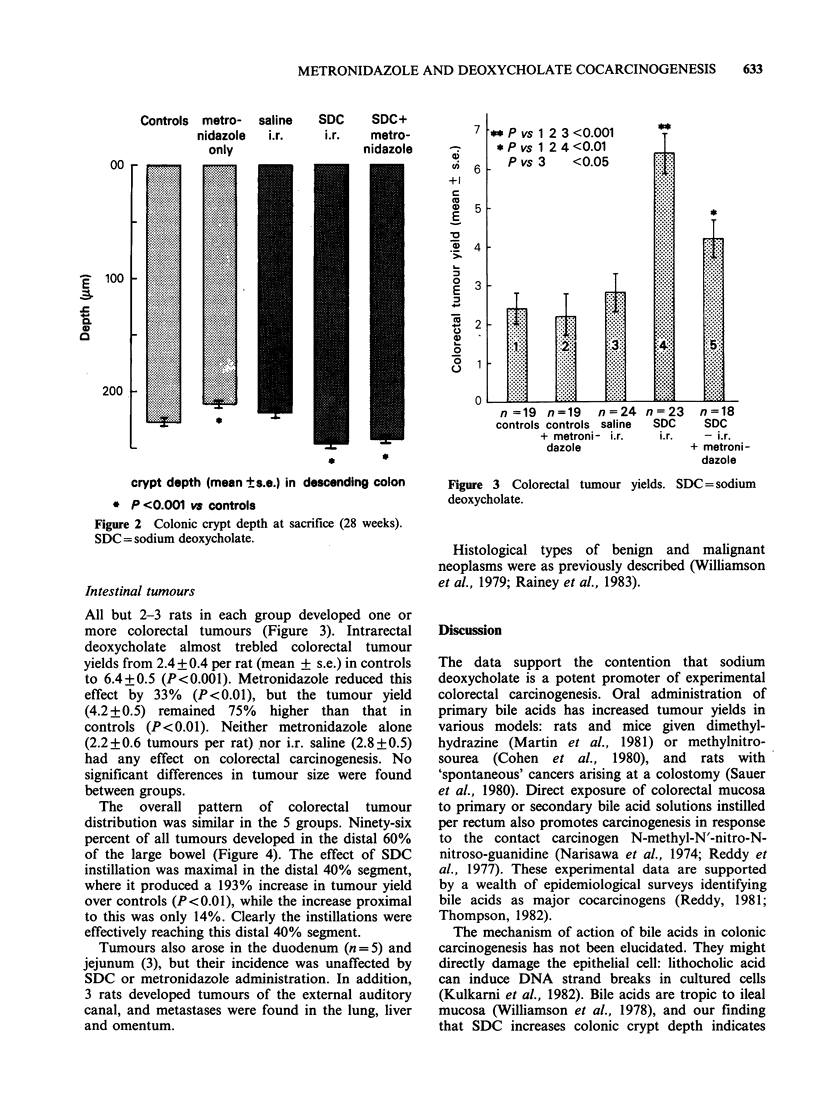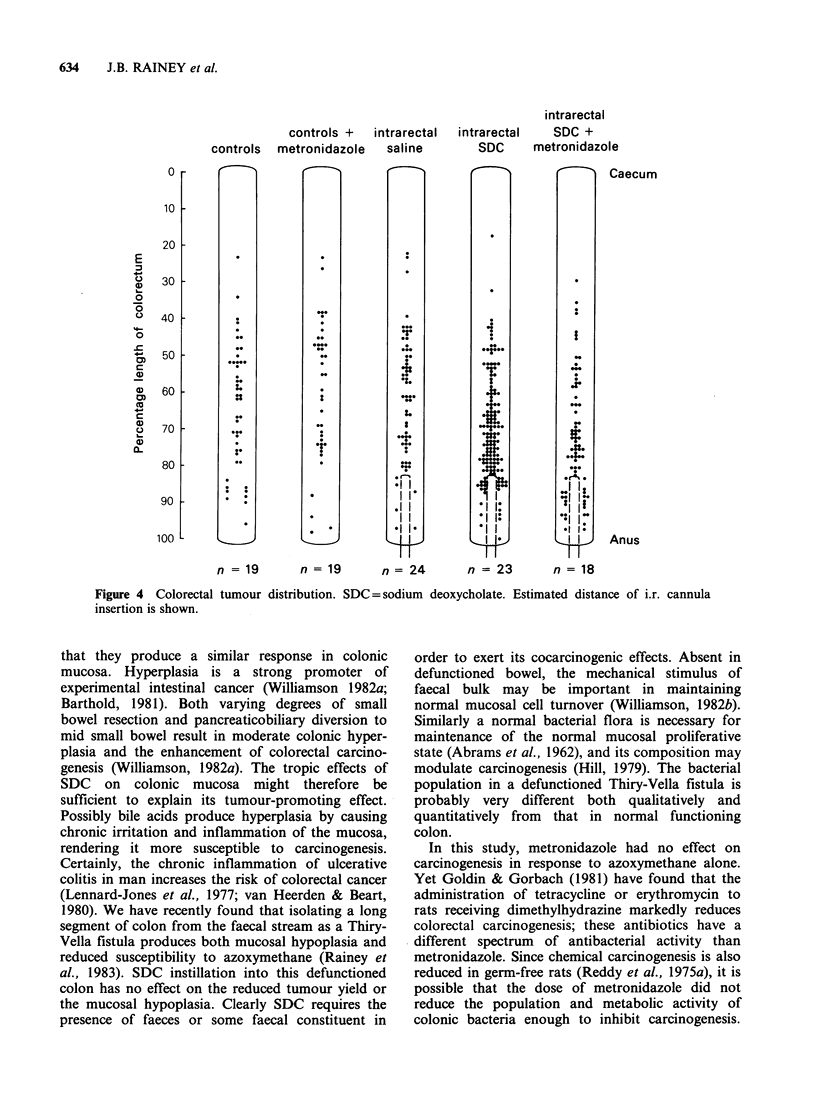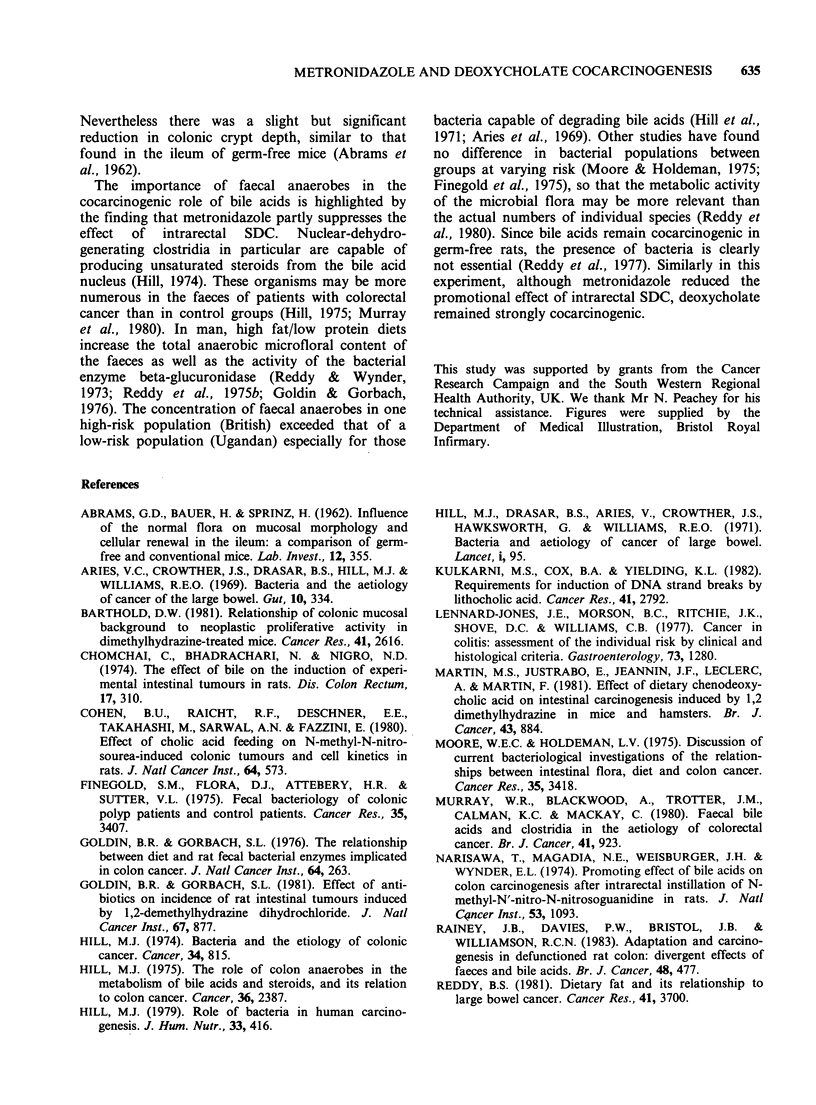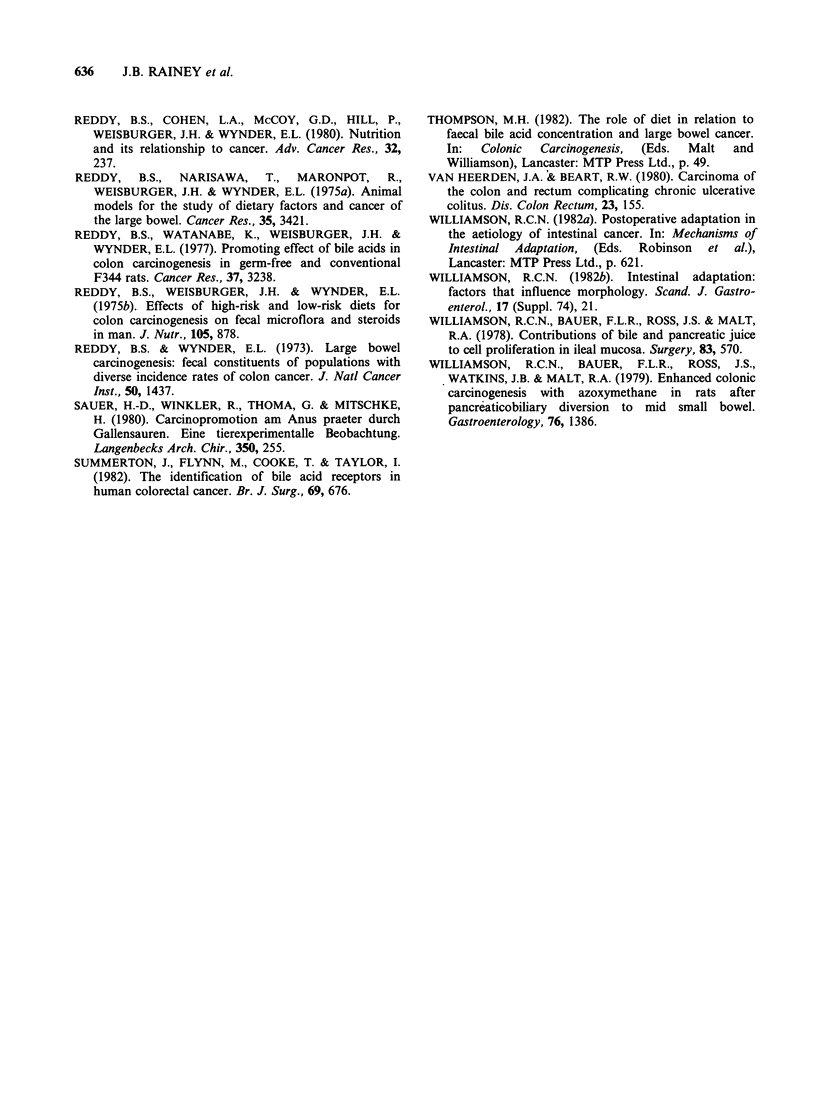# The cocarcinogenic effect of intrarectal deoxycholate in rats is reduced by oral metronidazole.

**DOI:** 10.1038/bjc.1984.98

**Published:** 1984-05

**Authors:** J. B. Rainey, M. Maeda, C. Williams, R. C. Williamson

## Abstract

Bile acids enhance colorectal carcinogenesis in animals and man, perhaps after degradation by faecal anaerobes. The promotional effect of sodium deoxycholate (SDC) and its relationship to bacteria was examined in male Sprague-Dawley rats (n = 115) which had received a 6-week course of azoxymethane (total dose 90 mg kg-1 s.c.) Two groups received 3 X weekly intrarectal (i.r.) instillations of N saline or 0.12 M SDC for 18 weeks. Another group received SDC i.r. plus metronidazole (22.5 mg kg-1) daily in the drinking water. Controls had no instillations or metronidazole alone. By 28 weeks SDC had increased mean colonic crypt depth by 9% (P less than 0.001), and had almost trebled colorectal tumour yields from 2.4 +/- 0.4 per rat (mean +/- s.e.) in controls to 6.4 +/- 0.5 (P less than 0.001). Tumour yields after SDC + metronidazole (4.2 +/- 0.5) remained 75% higher than in controls (P less than 0.01) but were 33% less than after SDC alone (P less than 0.01), and the increase in crypt depth was maintained at 7% (P less than 0.001). Neither metronidazole alone nor saline i.r. had any effect on tumour yield, but metronidazole alone reduced crypt depth by 9% (P less than 0.001). Deoxycholate is a potent cocarcinogen and also stimulates mucosal hyperplasia. Metronidazole reduces its tumour-promoting effect, suggesting that faecal anaerobes are important in bile acid cocarcinogenesis.


Br. J. Cancer (1984), **49**, 631–636

### The cocarcinogenic effect of intrarectal deoxycholate in rats is reduced by oral metronidazole

J.B. Rainey, M. Maeda, C. Williams & R.C.N. Williamson

*University Department of Surgery, Bristol Royal Infirmary, Bristol BS2 8HW, UK.*

**Summary** Bile acids enhance colorectal carcinogenesis in animals and man, perhaps after degradation by faecal anaerobes. The promotional effect of sodium deoxycholate (SDC) and its relationship to bacteria was examined in male Sprague-Dawley rats ($n=115$) which had received a 6-week course of azoxymethane (total dose $90\,mg\,kg^{-1}$ s.c.) Two groups received 3× weekly intrarectal (i.r.) instillations of N saline or 0.12 M SDC for 18 weeks. Another group received SDC i.r. plus metronidazole ($22.5\,mg\,kg^{-1}$) daily in the drinking water. Controls had no instillations or metronidazole alone. By 28 weeks SDC had increased mean colonic crypt depth by 9% ($P<0.001$), and had almost trebled colorectal tumour yields from $2.4 \pm 0.4$ per rat (mean ± s.e.) in controls to $6.4 \pm 0.5$ ($P<0.001$). Tumour yields after SDC + metronidazole ($4.2 \pm 0.5$) remained 75% higher than in controls ($P<0.01$) but were 33% less than after SDC alone ($P<0.01$), and the increase in crypt depth was maintained at 7% ($P<0.001$). Neither metronidazole alone nor saline i.r. had any effect on tumour yield, but metronidazole alone reduced crypt depth by 9% ($P<0.001$). Deoxycholate is a potent cocarcinogen and also stimulates mucosal hyperplasia. Metronidazole reduces its tumour-promoting effect, suggesting that faecal anaerobes are important in bile acid cocarcinogenesis.

Bile acids are strong candidates for the role of endogenous promoters of colorectal cancer (Reddy, 1981; Thompson, 1982). They bear a close steric resemblance to an established group of carcinogens, the polycyclic aromatic hydrocarbons. Human gut flora can cause partial aromatization of the steroid ring, and full conversion to 3-methylcholanthrene might be achieved by a series of chemical reactions (Hill, 1971). Faecal excretion of bile acids is greater in populations from high-risk countries (Western Europe, USA) as opposed to low-risk countries (Asia, Africa), and in meat eaters as opposed to vegetarians (Reddy & Wynder, 1973; Hill *et al.*, 1971; Aries *et al.*, 1969). Deoxycholic acid receptors have been identified in human colorectal cancers (Summerton *et al.*, 1982).

In theory, bile acids and their metabolites could either exert a direct mutagenic effect on the epithelial cell or act indirectly by altering the rate of mucosal cell proliferation and hence susceptibility to carcinogenesis. In the rat diversion of pancreaticobiliary secretions to mid small bowel enhances colorectal carcinogenesis (Chomchai *et al.*, 1974; Williamson *et al.*, 1979) and also stimulates marked adaptive hyperplasia of the ileum and moderate colonic hyperplasia (Williamson *et al.*, 1978). However, bile acids appear to be neither tropic nor cocarcinogenic to hypoplastic defunctioned colon (Rainey *et al.*, 1983). The composition of the colonic microbial flora is implicated as the key intermediary modulating the effect of luminal bile acids (Aries *et al.*, 1969; Hill, 1979). Anaerobes, in particular, may metabolise bile acids to yield products which are carcinogenic or cocarcinogenic. (Hill, 1974).

This experiment was designed to test the tropic and cocarcinogenic potential of sodium deoxycholate instilled directly into the large bowel of rats exposed to azoxymethane. In addition we examined the effect of oral metronidazole, an anaerobicide, in modifying this potential.

### Materials and methods

### *Experimental animals*

One hundred and fifteen male Sprague-Dawley rats (Olac SD, Bicester, Oxon) weighing 70–100 g were received into the animal house 1 week before the start of the experiment and were allocated to one of five groups (Figure 1). They were fed standard rat chow (Oxoid Breeding Diet; H C Styles & Co Ltd., Bewdley, Worcs) and water *ad libitum*. Animal quarters were lit in alternate 12-hourly cycles. Rats were weighed weekly throughout the experiment. All animals received weekly s.c. injections of azoxymethane (Ash Stevens Inc., Detroit, Michigan, USA) $15\,mg\,kg^{-1}$ for 6 weeks (Figure 1).

One week after the last injection of azoxymethane intrarectal (i.r.) instillations were carried out for the first time in groups 3–5. Colonic washout was not carried out and anaesthesia was unnecessary. An 18-gauge plastic i.v. cannula was

Correspondence: R.C.N. Williamson
Received 3 January 1984; accepted 24 January 1984.

© The Macmillan Press Ltd., 1984

**Figure 1** Experimental design. SDC = sodium deoxycholate. Numbers in each group at the start of the experiment and surviving until sacrifice are shown.

inserted through the anus to a distance of 5 cm in rats suitably restrained by an experienced handler. Rats in group 3 received 1 ml N saline. Groups 4 and 5 received 1 ml of 0.12 M sodium deoxycholate (SDC) prepared by dissolving 50 g SDC (Sigma Chemical Co., St Louis, USA) in 1 litre N saline; each 1 ml aliquot contained 0.05 g (120 $\mu$mol) of SDC. Instillations were carried out 3 times per week for 18 weeks (Figure 1).

In addition, rats in group 5 received metronidazole (22.5 mg kg$^{-1}$ rat day$^{-1}$; May & Baker Ltd., Dagenham, Essex) dissolved in the drinking water from the start of the instillations until the end of the experiment. Group 1 rats (controls) received neither i.r. instillations nor metronidazole, and those in group 2 received metronidazole alone (Figure 1).

*Autopsy specimens*

Rats were regularly examined for evidence of tumour development and were killed when moribund or at the end of 28 weeks. At autopsy the entire intestinal tract was excised. The following segments were thoroughly flushed with cold saline to remove all content: duodenum, jejunoileum and colorectum. The length of each segment was determined by suspension with a constant weight against a ruler. Segments were then opened, and the number, size and position of all tumours were recorded. After excision of the tumours the remaining bowel was blotted dry and weighed. The net weights of the caecum, liver, kidneys and spleen were also recorded. All tumours were fixed in 10% formalin before histological processing. Subsequently 5 $\mu$m sections were prepared for staining with haematoxylin and eosin.

In addition, a 1 cm specimen of colon was excised 5–6 cm from the anus, and similar histological specimens were prepared. The mean crypt depth was estimated by ocular micrometry of 10 perfectly-sectioned crypts per slide.

*Statistics*

Student's *t*-test was used for statistical analysis of the data.

### Results

*Mortality rate*

Eleven rats (10%) died before sacrifice either from colonic perforation during instillation (2 rats), haemorrhage secondary to duodenal or colonic cancer (2), haematuria (1), pneumonia (2), or cancer of the external auditory canal (1). In 3 rats that died during the early part of the experiment, the cause of death could not be determined. The yields of surviving animals at the end of the experiment are given in Figure 1.

*Body weight*

Rats in all groups gained weight steadily until week 25, after which weights remained constant until sacrifice 3 weeks later. Neither i.r. deoxycholate nor oral metronidazole had any consistent effect on body wt.

*Intestinal adaptation*

No differences between the groups were found in the lengths and weights of the duodenum, jejunoileum or colorectum, nor in the weights of the caecum, liver, kidneys or spleen.

The mean colonic crypt depth in controls was 226 ± 3 $\mu$m (mean ± s.e.) compared with 246 ± 3 $\mu$m in the SDC-irrigated group and 242 ± 3 $\mu$m in the SDC + metronidazole group ($P < 0.001$; Figure 2). Intrarectal saline had no effect on crypt depth compared with controls, but metronidazole alone produced a 9% decrease (211 ± 3 $\mu$m: $P < 0.001$).

**Figure 2** Colonic crypt depth at sacrifice (28 weeks). SDC = sodium deoxycholate.

**Figure 3** Colorectal tumour yields. SDC = sodium deoxycholate.

*Intestinal tumours*

All but 2–3 rats in each group developed one or more colorectal tumours (Figure 3). Intrarectal deoxycholate almost trebled colorectal tumour yields from 2.4 ± 0.4 per rat (mean ± s.e.) in controls to 6.4 ± 0.5 ($P$<0.001). Metronidazole reduced this effect by 33% ($P$<0.01), but the tumour yield (4.2 ± 0.5) remained 75% higher than that in controls ($P$<0.01). Neither metronidazole alone (2.2 ± 0.6 tumours per rat) nor i.r. saline (2.8 ± 0.5) had any effect on colorectal carcinogenesis. No significant differences in tumour size were found between groups.

The overall pattern of colorectal tumour distribution was similar in the 5 groups. Ninety-six percent of all tumours developed in the distal 60% of the large bowel (Figure 4). The effect of SDC instillation was maximal in the distal 40% segment, where it produced a 193% increase in tumour yield over controls ($P$<0.01), while the increase proximal to this was only 14%. Clearly the instillations were effectively reaching this distal 40% segment.

Tumours also arose in the duodenum ($n$ = 5) and jejunum (3), but their incidence was unaffected by SDC or metronidazole administration. In addition, 3 rats developed tumours of the external auditory canal, and metastases were found in the lung, liver and omentum.

Histological types of benign and malignant neoplasms were as previously described (Williamson *et al.*, 1979; Rainey *et al.*, 1983).

### Discussion

The data support the contention that sodium deoxycholate is a potent promoter of experimental colorectal carcinogenesis. Oral administration of primary bile acids has increased tumour yields in various models: rats and mice given dimethylhydrazine (Martin *et al.*, 1981) or methylnitrosourea (Cohen *et al.*, 1980), and rats with 'spontaneous' cancers arising at a colostomy (Sauer *et al.*, 1980). Direct exposure of colorectal mucosa to primary or secondary bile acid solutions instilled per rectum also promotes carcinogenesis in response to the contact carcinogen N-methyl-N′-nitro-N-nitroso-guanidine (Narisawa *et al.*, 1974; Reddy *et al.*, 1977). These experimental data are supported by a wealth of epidemiological surveys identifying bile acids as major cocarcinogens (Reddy, 1981; Thompson, 1982).

The mechanism of action of bile acids in colonic carcinogenesis has not been elucidated. They might directly damage the epithelial cell: lithocholic acid can induce DNA strand breaks in cultured cells (Kulkarni *et al.*, 1982). Bile acids are tropic to ileal mucosa (Williamson *et al.*, 1978), and our finding that SDC increases colonic crypt depth indicates

**Figure 4** Colorectal tumour distribution. SDC = sodium deoxycholate. Estimated distance of i.r. cannula insertion is shown.

that they produce a similar response in colonic mucosa. Hyperplasia is a strong promoter of experimental intestinal cancer (Williamson 1982*a*; Barthold, 1981). Both varying degrees of small bowel resection and pancreaticobiliary diversion to mid small bowel result in moderate colonic hyperplasia and the enhancement of colorectal carcinogenesis (Williamson, 1982*a*). The tropic effects of SDC on colonic mucosa might therefore be sufficient to explain its tumour-promoting effect. Possibly bile acids produce hyperplasia by causing chronic irritation and inflammation of the mucosa, rendering it more susceptible to carcinogenesis. Certainly, the chronic inflammation of ulcerative colitis in man increases the risk of colorectal cancer (Lennard-Jones *et al.*, 1977; van Heerden & Beart, 1980). We have recently found that isolating a long segment of colon from the faecal stream as a Thiry-Vella fistula produces both mucosal hypoplasia and reduced susceptibility to azoxymethane (Rainey *et al.*, 1983). SDC instillation into this defunctioned colon has no effect on the reduced tumour yield or the mucosal hypoplasia. Clearly SDC requires the presence of faeces or some faecal constituent in order to exert its cocarcinogenic effects. Absent in defunctioned bowel, the mechanical stimulus of faecal bulk may be important in maintaining normal mucosal cell turnover (Williamson, 1982*b*). Similarly a normal bacterial flora is necessary for maintenance of the normal mucosal proliferative state (Abrams *et al.*, 1962), and its composition may modulate carcinogenesis (Hill, 1979). The bacterial population in a defunctioned Thiry-Vella fistula is probably very different both qualitatively and quantitatively from that in normal functioning colon.

In this study, metronidazole had no effect on carcinogenesis in response to azoxymethane alone. Yet Goldin & Gorbach (1981) have found that the administration of tetracycline or erythromycin to rats receiving dimethylhydrazine markedly reduces colorectal carcinogenesis; these antibiotics have a different spectrum of antibacterial activity than metronidazole. Since chemical carcinogenesis is also reduced in germ-free rats (Reddy *et al.*, 1975*a*), it is possible that the dose of metronidazole did not reduce the population and metabolic activity of colonic bacteria enough to inhibit carcinogenesis.

Nevertheless there was a slight but significant reduction in colonic crypt depth, similar to that found in the ileum of germ-free mice (Abrams *et al.*, 1962).

The importance of faecal anaerobes in the cocarcinogenic role of bile acids is highlighted by the finding that metronidazole partly suppresses the effect of intrarectal SDC. Nuclear-dehydrogenerating clostridia in particular are capable of producing unsaturated steroids from the bile acid nucleus (Hill, 1974). These organisms may be more numerous in the faeces of patients with colorectal cancer than in control groups (Hill, 1975; Murray *et al.*, 1980). In man, high fat/low protein diets increase the total anaerobic microfloral content of the faeces as well as the activity of the bacterial enzyme beta-glucuronidase (Reddy & Wynder, 1973; Reddy *et al.*, 1975*b*; Goldin & Gorbach, 1976). The concentration of faecal anaerobes in one high-risk population (British) exceeded that of a low-risk population (Ugandan) especially for those bacteria capable of degrading bile acids (Hill *et al.*, 1971; Aries *et al.*, 1969). Other studies have found no difference in bacterial populations between groups at varying risk (Moore & Holdeman, 1975; Finegold *et al.*, 1975), so that the metabolic activity of the microbial flora may be more relevant than the actual numbers of individual species (Reddy *et al.*, 1980). Since bile acids remain cocarcinogenic in germ-free rats, the presence of bacteria is clearly not essential (Reddy *et al.*, 1977). Similarly in this experiment, although metronidazole reduced the promotional effect of intrarectal SDC, deoxycholate remained strongly cocarcinogenic.

This study was supported by grants from the Cancer Research Campaign and the South Western Regional Health Authority, UK. We thank Mr N. Peachey for his technical assistance. Figures were supplied by the Department of Medical Illustration, Bristol Royal Infirmary.

**References**

ABRAMS, G.D., BAUER, H. & SPRINZ, H. (1962). Influence of the normal flora on mucosal morphology and cellular renewal in the ileum: a comparison of germ-free and conventional mice. *Lab. Invest.*, **12**, 355.

ARIES, V.C., CROWTHER, J.S., DRASAR, B.S., HILL, M.J. & WILLIAMS, R.E.O. (1969). Bacteria and the aetiology of cancer of the large bowel. *Gut*, **10**, 334.

BARTHOLD, D.W. (1981). Relationship of colonic mucosal background to neoplastic proliferative activity in dimethylhydrazine-treated mice. *Cancer Res.*, **41**, 2616.

CHOMCHAI, C., BHADRACHARI, N. & NIGRO, N.D. (1974). The effect of bile on the induction of experimental intestinal tumours in rats. *Dis. Colon Rectum*, **17**, 310.

COHEN, B.U., RAICHT, R.F., DESCHNER, E.E., TAKAHASHI, M., SARWAL, A.N. & FAZZINI, E. (1980). Effect of cholic acid feeding on N-methyl-N-nitrosourea-induced colonic tumours and cell kinetics in rats. *J. Natl Cancer Inst.*, **64**, 573.

FINEGOLD, S.M., FLORA, D.J., ATTEBERY, H.R. & SUTTER, V.L. (1975). Fecal bacteriology of colonic polyp patients and control patients. *Cancer Res.*, **35**, 3407.

GOLDIN, B.R. & GORBACH, S.L. (1976). The relationship between diet and rat fecal bacterial enzymes implicated in colon cancer. *J. Natl Cancer Inst.*, **64**, 263.

GOLDIN, B.R. & GORBACH, S.L. (1981). Effect of antibiotics on incidence of rat intestinal tumours induced by 1,2-demethylhydrazine dihydrochloride. *J. Natl Cancer Inst.*, **67**, 877.

HILL, M.J. (1974). Bacteria and the etiology of colonic cancer. *Cancer*, **34**, 815.

HILL, M.J. (1975). The role of colon anaerobes in the metabolism of bile acids and steroids, and its relation to colon cancer. *Cancer*, **36**, 2387.

HILL, M.J. (1979). Role of bacteria in human carcinogenesis. *J. Hum. Nutr.*, **33**, 416.

HILL, M.J., DRASAR, B.S., ARIES, V., CROWTHER, J.S., HAWKSWORTH, G. & WILLIAMS, R.E.O. (1971). Bacteria and aetiology of cancer of large bowel. *Lancet*, **i**, 95.

KULKARNI, M.S., COX, B.A. & YIELDING, K.L. (1982). Requirements for induction of DNA strand breaks by lithocholic acid. *Cancer Res.*, **41**, 2792.

LENNARD-JONES, J.E., MORSON, B.C., RITCHIE, J.K., SHOVE, D.C. & WILLIAMS, C.B. (1977). Cancer in colitis: assessment of the individual risk by clinical and histological criteria. *Gastroenterology*, **73**, 1280.

MARTIN, M.S., JUSTRABO, E., JEANNIN, J.F., LECLERC, A. & MARTIN, F. (1981). Effect of dietary chenodeoxycholic acid on intestinal carcinogenesis induced by 1,2 dimethylhydrazine in mice and hamsters. *Br. J. Cancer*, **43**, 884.

MOORE, W.E.C. & HOLDEMAN, L.V. (1975). Discussion of current bacteriological investigations of the relationships between intestinal flora, diet and colon cancer. *Cancer Res.*, **35**, 3418.

MURRAY, W.R., BLACKWOOD, A., TROTTER, J.M., CALMAN, K.C. & MACKAY, C. (1980). Faecal bile acids and clostridia in the aetiology of colorectal cancer. *Br. J. Cancer*, **41**, 923.

NARISAWA, T., MAGADIA, N.E., WEISBURGER, J.H. & WYNDER, E.L. (1974). Promoting effect of bile acids on colon carcinogenesis after intrarectal instillation of N-methyl-N′-nitro-N-nitrosoguanidine in rats. *J. Natl Cancer Inst.*, **53**, 1093.

RAINEY, J.B., DAVIES, P.W., BRISTOL, J.B. & WILLIAMSON, R.C.N. (1983). Adaptation and carcinogenesis in defunctioned rat colon: divergent effects of faeces and bile acids. *Br. J. Cancer*, **48**, 477.

REDDY, B.S. (1981). Dietary fat and its relationship to large bowel cancer. *Cancer Res.*, **41**, 3700.

REDDY, B.S., COHEN, L.A., McCOY, G.D., HILL, P., WEISBURGER, J.H. & WYNDER, E.L. (1980). Nutrition and its relationship to cancer. *Adv. Cancer Res.*, **32**, 237.

REDDY, B.S., NARISAWA, T., MARONPOT, R., WEISBURGER, J.H. & WYNDER, E.L. (1975*a*). Animal models for the study of dietary factors and cancer of the large bowel. *Cancer Res.*, **35**, 3421.

REDDY, B.S., WATANABE, K., WEISBURGER, J.H. & WYNDER, E.L. (1977). Promoting effect of bile acids in colon carcinogenesis in germ-free and conventional F344 rats. *Cancer Res.*, **37**, 3238.

REDDY, B.S., WEISBURGER, J.H. & WYNDER, E.L. (1975*b*). Effects of high-risk and low-risk diets for colon carcinogenesis on fecal microflora and steroids in man. *J. Nutr.*, **105**, 878.

REDDY, B.S. & WYNDER, E.L. (1973). Large bowel carcinogenesis: fecal constituents of populations with diverse incidence rates of colon cancer. *J. Natl Cancer Inst.*, **50**, 1437.

SAUER, H.-D., WINKLER, R., THOMA, G. & MITSCHKE, H. (1980). Carcinopromotion am Anus praeter durch Gallensauren. Eine tierexperimentalle Beobachtung. *Langenbecks Arch. Chir.*, **350**, 255.

SUMMERTON, J., FLYNN, M., COOKE, T. & TAYLOR, I. (1982). The identification of bile acid receptors in human colorectal cancer. *Br. J. Surg.*, **69**, 676.

THOMPSON, M.H. (1982). The role of diet in relation to faecal bile acid concentration and large bowel cancer. In: *Colonic Carcinogenesis*, (Eds. Malt and Williamson), Lancaster: MTP Press Ltd., p. 49.

VAN HEERDEN, J.A. & BEART, R.W. (1980). Carcinoma of the colon and rectum complicating chronic ulcerative colitus. *Dis. Colon Rectum*, **23**, 155.

WILLIAMSON, R.C.N. (1982*a*). Postoperative adaptation in the aetiology of intestinal cancer. In: *Mechanisms of Intestinal Adaptation*, (Eds. Robinson *et al.*), Lancaster: MTP Press Ltd., p. 621.

WILLIAMSON, R.C.N. (1982*b*). Intestinal adaptation: factors that influence morphology. *Scand. J. Gastroenterol.*, **17** (Suppl. 74), 21.

WILLIAMSON, R.C.N., BAUER, F.L.R., ROSS, J.S. & MALT, R.A. (1978). Contributions of bile and pancreatic juice to cell proliferation in ileal mucosa. *Surgery*, **83**, 570.

WILLIAMSON, R.C.N., BAUER, F.L.R., ROSS, J.S., WATKINS, J.B. & MALT, R.A. (1979). Enhanced colonic carcinogenesis with azoxymethane in rats after pancreaticobiliary diversion to mid small bowel. *Gastroenterology*, **76**, 1386.